# Cultivation and Differentiation of Encapsulated hMSC-TERT in a Disposable Small-Scale Syringe-Like Fixed Bed Reactor

**DOI:** 10.2174/1874120700701010064

**Published:** 2007-10-29

**Authors:** Christian Weber, Sebastian Pohl, Ralf Pörtner, Christine Wallrapp, Moustapha Kassem, Peter Geigle, Peter Czermak

**Affiliations:** 1 Institute of Biopharmaceutical Technology, University of Applied Sciences Giessen-Friedberg, Giessen-Germany; 2 Institute of Bioprocess and Biosystems Engineering, University of Technology, Hamburg-Germany; 3 CellMed AG, Alzenau, Germany; 4 Department of Endocrinology and Metabolism, University Hospital of Odense, Odense-Denmark; 5 Department of Chemical Engineering, Kansas State University, Manhattan KS-USA

**Keywords:** Adipogenic differentiation, alginate, disposable fixed bed reactor, mesenchymal stem cells, oxygen measurement, syringe.

## Abstract

The use of commercially available plastic syringes is introduced as disposable small-scale fixed bed bioreactors for the cultivation of implantable therapeutic cell systems on the basis of an alginate-encapsulated human mesenchymal stem cell line. The system introduced is fitted with a noninvasive oxygen sensor for the continuous monitoring of the cultivation process. Fixed bed bioreactors offer advantages in comparison to other systems due to their ease of automation and online monitoring capability during the cultivation process. These benefits combined with the advantage of single-use make the fixed bed reactor an interesting option for GMP processes. The cultivation of the encapsulated cells in the fixed bed bioreactor system offered vitalities and adipogenic differentiation similar to well-mixed suspension cultures.

## INTRODUCTION

In research and pharmaceutical industry, many small-scale bioreactors such as stirred tanks, spinner and tissue culture flasks, and rocked bags are used for the cultivation of animal cells [1,2]. The latter three are available as disposable systems, which offer many advantages including the avoidance of cleaning procedures and availability as sterilized ready-to-use units [3]. These characteristics allow their use in GMP processes as well as in the optimization of system variables at low-scale [4, 5].

For the cultivation of immobilized cells, fixed bed bioreactors may be used. They offer easy control and automation of the process, low shear stresses, and medium conditioning in separate vessels [6, 7]. The use of commercially available plastic syringes as small-scale, disposable fixed bed reactors is introduced and demonstrated by cultivation of an alginate-encapsulated stem cell line. Single-use syringes are commonly available from 1 to 100 ml as sterile packed single units, offering the benefit of disposable bioreactor systems that can be adapted to different cultivation processes.

The alginate-encapsulated stem cell line is traded as CellBeads^©^ (patent number: US 6,465,226) by the CellMed AG (Alzenau, Germany). hMSC-TERT are human mesenchymal stem cells modified by transfection with a telomerase activity to increase the number of achievable population doublings [8, 9]. CellBeads^©^ are implantable therapeutic cell systems which possess the potential to counteract endocrine deficiencies *in vivo* [10]. The CellBeads^©^ consist of an inner alginate-cell core with a diameter of about 400 µm surrounded by an alginate layer with a diameter of about 640 µm. Before implantation, the CellBeads^©^ need to be differentiated.

In the special case of cell therapy the cells have to be differentiated prior to use. Bioreactors should maintain the vitality of cells by providing sufficient nutrient and oxygen concentrations within all areas of the vessel. Furthermore, bioreactors should be easy to handle and maintain sterility during cultivation [11]. The possibilities for automation and monitoring of the cultivation process are important issues too.

The above claims relative to a bioreactor system were used as criteria for verification of plastic syringes as disposable fixed bed reactors for the cultivation of implantable cell systems (CellBeads^©^).

## MATERIALS AND METHODS

All chemicals were obtained from Sigma-Aldrich (Deisenhofen, Germany) unless otherwise indicated.

### EDTA Stock Solution (0.5 M)

For preparation of a 0.5 M EDTA (ethylenediamine tetraacetic acid) stock solution, 3.84 g EDTA were dissolved in 50 ml deionised water. The pH-value was adjusted to 8.0 by titration of HCl. The prepared solution was sterilized by filtration (0.2 µm) and thereby storable at ambient conditions.

### Lysis Buffer

The lysis buffer was used to disintegrate the CellBeads^©^ prior to the vitality staining with trypan blue. The principle of the lysis buffer is the depolymerisation of the alginate layer through the formation of a complex with the bivalent cations. The break up of the alginate causes the release of the immobilized cells. For the preparation of this buffer, PBS (phosphate buffered saline, Biowest, Nuaille, France) was supplemented with 10 mM EDTA (2 ml EDTA stock solution per 98 ml PBS buffer) and 0.1% BSA.

### Tris-HCl Buffer (100 mM)

Tris buffer was used for the preparation of SYBR Green solution. 3.14 g Tris were dissolved in 250 ml deionised water. The pH-value was adjusted to 8.0 using HCl. The prepared solution was autoclaved and stored at room temperature.

### SYBR Green and Propidium Iodide Solution

The fluorescence dyes SYBR Green and propidium iodide were used for visualization of the vitality of the encapsulated cells. A 20-fold concentrated stock solution of SYBR Green was prepared using the original 10.000 fold SYBR Green + DMSO solution. The stock solution is stable at -20°C for up to one year. The working solution was prepared by adding 500 µl SYBR Green stock solution and 500 µl EDTA solution to 1500 µl Tris-HCl buffer. For preparation of the propidium iodide solution, 5 ml PBS were added to 25 mg propidium iodide (Sigma-Aldrich). The solution is stable in an opaque bottle at 4°C for up to 6 months.

### Nile Red Solution

Nile red is a fluorescent lipid staining dye and used to verify the adipogenic differentiation status of the cells [12]. A 1 mg/ml stock solution of nile red in ethanol was diluted in PBS to a final concentration of 1 µg/ml and clarified by filtration (0.22 µm) prior to use.

### Culture Medium

The CellBeads^©^ were cultured in EMEM (minimal essential medium with Earle’s salts, Biochrom AG, Berlin, Germany), supplemented with 10% BGS (Bovine Growth Serum, Thermo Fisher Scientific, Schwerte, Germany), 100 U/ml penicillin and 100 µg/ml streptomycin. The medium was used for cultivation without adipogenic differentiation.

### Induction Medium

The induction medium was used to induce the adipogenic differentiation of the encapsulated cells.

The induction medium consisted of DMEM-HG (Dulbecco’s modified Eagle medium - high glucose, Biowest, Nuaille, France), which was supplemented with 10% BGS, 100 U/ml penicillin and 100 µg/ml streptomycin (Sigma-Aldrich), 1 μM dexamethason (Sigma-Aldrich), 0.2 mM indomethacin (Sigma-Aldrich), 0.01 mg/ml insulin (Sigma-Aldrich) and 0.5 mM 3-isobutyl-1-methyl-xanthin (Sigma-Aldrich).

### Maintenance Medium

DMEM-HG supplemented with 10% BGS, 10 mg/l insulin, 100 U/ml penicillin and 100 µg/ml streptomycin were used as a culture medium between the induction phases of adipogenic differentiation.

### CellBeads^©^

The CellBeads^©^ were supplied in a frozen cryo vial. Prior to use they were thawed by placing the cryo vial in a 37°C water bath for 1-2 minutes. Afterwards, the CellBeads^©^ were transferred to a 25-cm^2^ tissue culture flask containing 20 ml conditioned culture medium, cultured for 1 hour at 37°C in a humidified 5% CO_2_ incubator and subsequently introduced into the fixed bed bioreactor system.

### Fixed Bed Bioreactor System

**Fig. (2):**
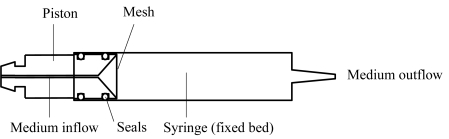
Syringe and piston drawn in the assembled condition.

**Fig. (3):**
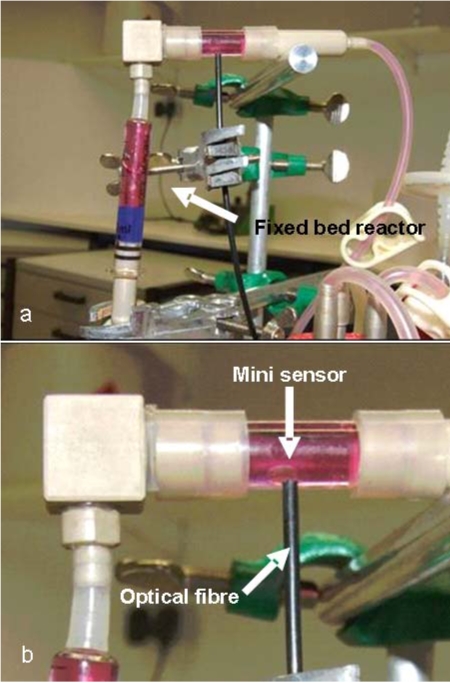
Handcrafted oxygen measurement chamber with integrated oxygen mini sensor for the measuring of the oxygen concentration in the medium outflow.

The core of the fixed bed cultivation system is the reactor which consists of a commercially available single-use plastic syringe and a special lathed piston, which enables the perfusion of the reactor and the embedding of the package (Fig. **2** and **3**). The funnel-shaped inflow area forces a uniform velocity profile upward at the inflow boundary of the fixed bed. The prototype of the piston is made of biocompatible polyetheretherketone (PEEK), covered at the top with a stainless steel mesh with an aperture size of 100 µm to retain the CellBeads^©^. Two O-rings made of the autoclavable Viton ^©^ serve as seals between syringe and piston.

A schematic of the experimental setup, including the small-scale fixed bed reactor (volume: 3 ml, diameter: 9 mm) and the periphery, is illustrated in Fig. (**4**). The system was perfused using a precision peristaltic pump (IPC-8, Ismatec, Glattbrugg, Switzerland), which enabled small volume flows with reduced pulsation. Two 250 ml flasks (Duran flask, Schott AG, Mainz, Germany), equipped with sterile filters (Midisart 0.22 µm, Sartorius AG, Goettingen, Germany) to maintain pressure equilibrium, were used as conditioning and waste vessels, respectively. A self-made measurement chamber, consisting of an oxygen mini sensor (PreSens, Regensburg, Germany) inserted into a glass tube fitted with PEEK hose connectors, enabled the noninvasive monitoring of the dissolved oxygen in the medium outflow (Fig. **3**). The mini sensor consists of a glass disc coated by a luminescent dye. Molecular oxygen caused a quenching of the luminescence and the oxygen dependant signal was measured *via* optical fibre (Fibox3, Presens) [13, 14].

### Cultivation of the CellBeads^©^

After autoclaving (121°C, 20 minutes), the conditioning flask was filled with medium (EMEM + 10% BGS), which was circulated by pumping to fill the tubings and to remove air bubbles. The system was placed in a humidified incubator (37°C, 5% CO_2_; Galaxy B, RS Biotech, Alloa, UK) for 1 hour to allow conditioning of the medium. Afterwards, the reactor chamber was opened by removing the piston to insert the CellBeads^©^ (1 ml package volume containing about 4500 CellBeads^©^, 2100 cells per CellBead^©^) and then closed by inserting the piston back into the syringe. During this procedure, the influx and efflux tubings were pinched to avoid a back flow of medium. All steps after autoclaving were carried out under sterile conditions.

The whole system except the peristaltic pump was placed in the incubator during cultivation. The medium flow was adjusted to 0,5 ml/min, leading to a dissolved oxygen concentration in the outflow of 78-86% of air saturation. The inflow was saturated with oxygen. The medium was completely changed every 3-4 days. The CellBeads^©^ were cultured for 200 and 500 hours, respectively. In each case, two cultivation runs were performed. Additionally, cultivations of 100 µl CellBeads^©^ in 25-cm^2^ tissue culture flasks with the same medium (20 ml) and medium changing intervals were executed as a reference. To ensure a homogeneous nutrient and oxygen concentration profile in the CellBead-medium suspension, the tissue culture flasks were positioned on an orbital shaker (30 rpm) and placed in the incubator. In addition, orbital shaking increased the oxygen transfer into the medium and thus supported optimized culture conditions for the reference CellBeads^©^ [15]. After cultivation the CellBeads^©^ were analysed for vitality.

### Vitality of the CellBeads^©^ - SYBR Green and Propidium Iodide Staining

SYBR Green and propidium iodide are fluorescence dyes which intercalate between the double helix of nucleic acids. SYBR Green can pass through the membrane of viable cells whereas propidium iodide is only able to enter necrotic cells with disintegrated membranes [16].

In each case, 100 µl CellBeads^©^ were transferred into 6-well cell culture plates. After adding 200 µl PBS, 10 µl propidium iodide and lastly 20 µl SYBR Green working solutions, the samples were cultured for 5 minutes in the dark. Pictures were taken for evaluation using a fluorescence microscope (Eclipse 80i, Nikon, Tokyo, Japan) at an excitation wavelength of 488 nm.

### Vitality of the Cell-Beads^©^ – Bead Lysis and Trypan Blue Staining

For a quantitative evaluation of the vitality of the CellBeads^©^ the alginate capsule and matrix was dissolved using the EDTA containing lysis buffer.

500 µl CellBeads^©^ were transferred into a 50 ml plastic tube and washed with PBS twice to remove traces of medium. Afterwards, 20 ml lysis buffer and 3 ml trypsine (Biowest, Nuaille, France) were added. After incubation for 20 minutes in a humidified incubator (37°C, 5% CO2), the suspension was resuspended 20-30 times to induce a certain sheer stress that causes the complete disintegration of the CellBeads^©^. Subsequently, 100 µl cell suspension were mixed with 100 µl trypan blue and incubated at ambient conditions for about 5 minutes. The number of vital cells and number of blue-stained dead cells were determined using a phase contrast microscope (DMIL, Leica AG, Wetzlar, Germany) by means of a Neubauer counting chamber.

### Adipogenic Differentiation of the CellBeads^©^

Additional adipogenic cultivations were performed to investigate the differentiation ability of the CellBeads^©^ during the cultivation process in the disposable syringe fixed bed reactor.

The CellBeads^©^ were cultured using the setup described above but instead of normal cultivation, medium induction and maintanance medium were used. Induction medium was applied for 3 days, followed by 4 days cultivation with maintenance medium. This cycle was repeated 3 times. After each medium change, the first 20 ml were discarded by means of perfusion of the system to avoid a mixture of the two media in the tubes. Because of the higher sodium bicarbonate content in DMEM, the CO_2_ concentration in the incubator was increased to 10 %. Additional reference cultivations in 25-cm^2^ tissue culture flasks were executed.

### Nile Red Staining

The nile red staining was performed after the adipogenic differentiation cultivation to verify the adipogenic differentiation of the CellBeads^©^.

To verify adipogenic differentiation about 50 CellBeads^©^ were transferred into a 6-well cell culture plate, rinsed three times with PBS after medium removal, fixed by the addition of 1ml methanal and incubated for one hour. Afterwards, the methanal was removed, followed by three washing steps with PBS. The subsequent incubation with 400 µl nile red solution for one hour was carried out in the dark. Pictures were taken using a fluorescence microscope (Eclipse 80i ) at an excitation wavelength of 550 nm.

## RESULTS AND DISCUSSION

**Fig. (5):**
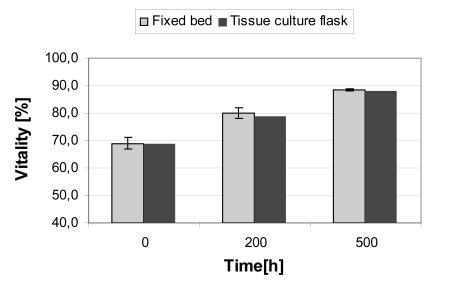
Vitality of the CellBeads^©^, cultured in the fixed bed reactor and tissue culture flasks. The vitalities were determined after lysis of the alginate capsules by the trypan blue exclusion method. The data (fixed bed) represents the mean ± standard deviation of two cultivations.

The CellBeads^©^ had an initial vitality of about 70%. The cultivation in both systems, the fixed bed reactor and the shaken, well-mixed tissue culture flasks, resulted in a similar increase of vitality. After a cultivation period of 200 hours, the vitality rose to about 80% and after 500 hours to 88% (Fig. **5**). The increasing vitality can be explained by a degradation of dead cells, based on the assumption that the cells in the CellBeads^©^ are non-proliferating. The similar vitality in both systems can be attributed to good nutritional supply within the entire fixed bed as well as the lack of additional apoptosis or necrosis during the cause of the cultivation.

**Fig. (6):**
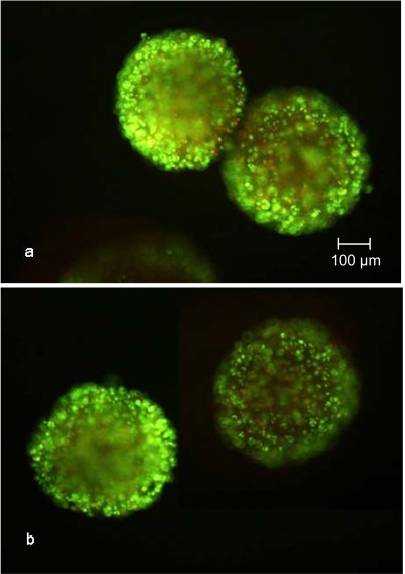
SYBR Green and propidium iodide stained CellBeads^©^ after 100 hours cultivation in the fixed bed reactor **(a)** and tissue culture flask **(b)**. Independent of the cultivation method, many necrotic cells, indicated by red luminescence, are visible.

**Fig. (7):**
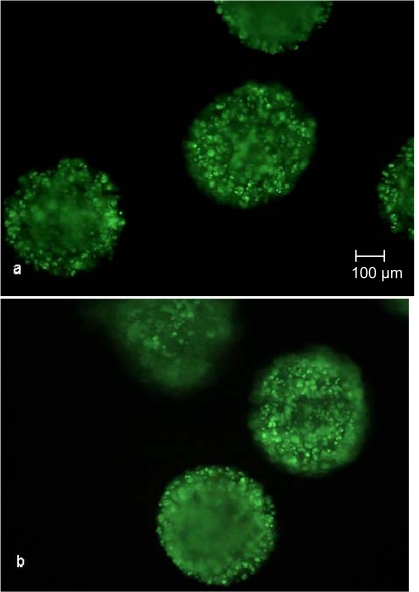
SYBR Green and propidium iodide staining after 500 h cultivation in the fixed bed reactor  **(a)** and tissue culture flask  **(b)**. No red luminescent necrotic cells are observable.

The increasing vitality during the cultivation, determined as described above, has been confirmed by the images taken after SYBR Green and propidium iodide staining (Fig. **6** and **7**). The number of propidium iodide stained dead cells decreased with increasing cultivation time. Whether the dead cells were necrotic or in the early state of apoptosis can not be stated, but it is more likely that the cells were in apoptosis because of the cultivation time-dependent decrease of propidium iodide stainable DNA.

However, as can seen in Fig. (**7**), no necrotic cells are detectable, thereby indicating a higher vitality compared to the trypan blue staining. Either cells inside the core of the CellBead^©^ are necrotic yet not visible because of the opacity of the beads, or the lysis process of the alginate capsule and matrix prior to trypan blue staining caused a decrease in vitality. The latter explanation would mean a higher vitality than 88%. Nevertheless, no difference between the cultivation in the fixed bed reactor and the tissue culture flasks has been found and thus no negative influence can be determined, such as sheer stresses induced by medium flow or bad percolation of certain areas of the fixed bed.

**Fig. (8):**
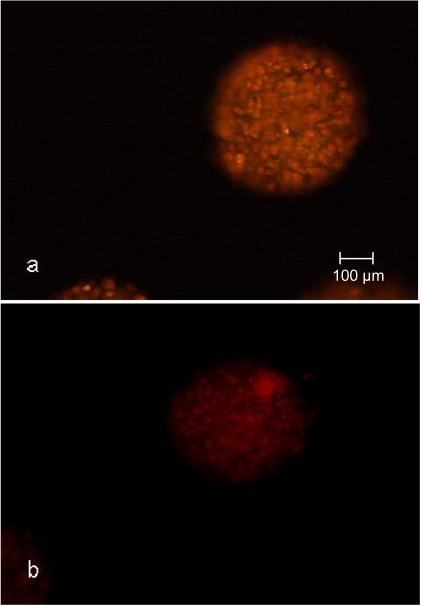
Nile red staining of CellBeads^©^ cultured under adipogenic  **(a)** and unstimulated non-adipogenic **(b)** conditions in the fixed bed reactor.

**Fig. (9):**
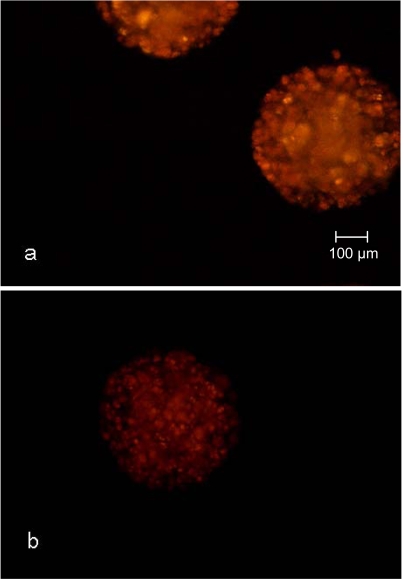
Nile red staining of CellBeads^©^ cultured under adipogenic  **(a)** and unstimulated non-adipogenic  **(b)** conditions in shaken tissue culture flasks.

Another criteria used for the qualification of syringes as disposable small-scale fixed bed reactors for the cultivation of encapsulated cells was the adipogenic differentiation potential during the cultivation. The nile red staining of the CellBeads^©^ after the three-week differentiation cultivation revealed no difference between the cultivation of CellBeads^©^ in the fixed bed reactor and tissue culture flasks (Fig. **8** and **9**). The higher fluorescence intensity (Fig. **8a**) and **9a**) of adipogenic cultured CellBeads^©^ compared to the unstimulated reference cultures (Fig. **8b**) and **9b**) points towards an enrichment in lipid content, thus indicating an adipogenic differentiation of the cells [17, 18].

Both the oxygen measurement at the outlet and the insecurity of the inflow oxygen concentration at the inlet of the reactor facilitated the monitoring of the cultivation process as well as the condition of the cells, enabling the determination of oxygen uptake kinetics. In Fig. (**10**), the oxygen profile of the outflow during adipogenic differentiation cultivation of the CellBeads^©^ demonstrates the oxygen measuring system.

During the cultivation of immobilized cells such as CellBeads^©^ in the fixed bed reactor, volume flow can be controlled by measuring the oxygen concentration in the outflow. Conditioning of the medium in the conditioning vessel is also possible. In the special case of the investigated CellBeads^©^, another benefit is apparent: because of the implantation of the CellBeads^©^ as biopharmaceutical cell systems *via* injection, replacement of the original syringe position with a special lathed piston allows the use of the disposable syringe bioreactor as an implantation instrument. This increases convenience since the CellBeads^©^ have not to be filled in a second device, thus reducing the risk of contamination due to fewer handling steps. Moreover, previously required washing steps for removal of culture medium can be performed automatically in the syringe reactor.

In addition to an easily realized automation, the benefits of the disposable syringe-like fixed bed system for the general use of cell cultivation or the cultivation of immobilized cell systems are lower costs, ready availability, easy handling, no cleaning steps, purchase ability as a sterile product and transparency for a visual monitoring of the fixed bed.

GMP guidelines require any system designed for single-use to have all parts in contact with the medium to be disposable. Therefore, the piston with the medium inflow connector, the mesh for package retainment as well as the measurement chamber with minisensor interfaced at the chamber outflow must be manufactured as single-use, sterile packaged items. Reduced manufacturing expenses may be achieved by choosing an appropriate synthetic material and a tight fitting of the piston to the syringe diameter, whereby avoiding the two O-ring seals. Moreover, the steel sieve used for the prototype may be replaced by a plastic sieve, preferably welded to the piston. The mesh aperture may be customized according to user demands. After integration of disposable oxygen or pH minisensors into a single-use measuring chamber and sterile packaging, this device may be manufactured by the supplier of the non-invasive oxygen and pH measurement system (Presens). Sterile packaged, single-use bottles, tubes, sterile filters and connectors are available from various manufacturers in a variety of designs and thus no problems related to the design of the disposable peripherals of the syringe-based fixed bed reactor are expected.

The measurement of oxygen enables a control of volume flow and through the use of a second measurement chamber at the medium inlet, both the calculation of oxygen uptake rates and an estimation of the oxygen concentration profile along the reactor axis is possible. Moreover, by measuring the oxygen concentration at the medium inlet the efficiency of oxygen transfer in the conditioning vessel may be determined.

## CONCLUSION

The qualification of disposable plastic syringes as small-scale single-use fixed bed reactors for the cultivation of encapsulated cells was investigated by cultivation and adipogenic differentiation of alginate capsuled hMSC-TERT (CellBeads^©^). Compared to the reference cultures in shaken 25-cm^2^ tissue culture flasks, no disadvantage concerning the viability and differentiation potential were detected. As shown in this study, no drawbacks concerning automation ability, low costs and availability as sterile products can be attributed to syringes as fixed bed reactors.

## Figures and Tables

**Fig. (1):**
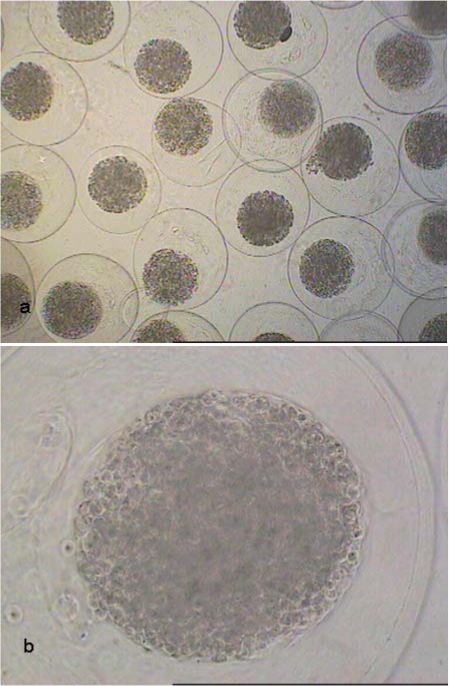
Light-microscopical images of CellBeads^©^ at a magnification of 40x **(a)** and 200x **(b)**.

**Fig. (4):**
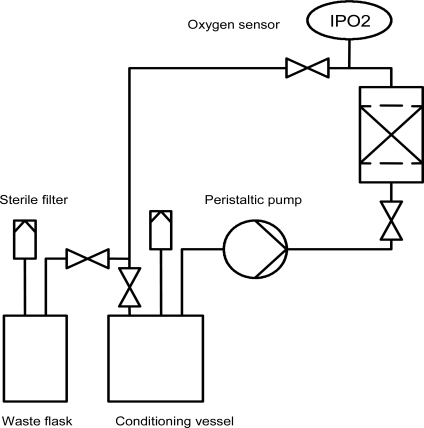
Schematic of the fixed bed reactor system and the corresponding periphery.

**Fig. (10):**
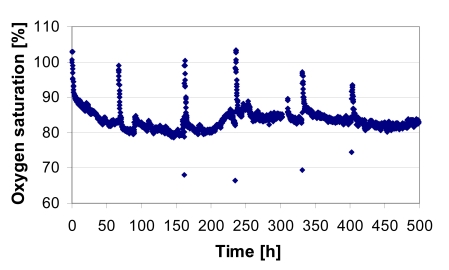
Oxygen saturation concentration at the medium outlet during the adipogenic differentiation cultivation. The peaks are due to the medium changes.
